# Common synonymous variants in *ABCA4* are protective for chloroquine induced maculopathy (toxic maculopathy)

**DOI:** 10.1186/s12886-015-0008-0

**Published:** 2015-03-06

**Authors:** Felix Grassmann, Richard Bergholz, Julia Mändl, Herbert Jägle, Klaus Ruether, Bernhard HF Weber

**Affiliations:** Institute of Human Genetics, University of Regensburg, Franz-Josef-Strauss-Allee 11, 93053 Regensburg, Germany; Charité Augenklinik Campus Virchow-Klinikum, Augustenburger Platz 1, 13353 Berlin, Germany; Department of Ophthalmology, University Hospital Regensburg, Franz-Josef-Strauss-Allee 11, 93053 Regensburg, Germany; Augenklinik, Sankt Gertrauden-Krankenhaus, Paretzer Strasse 12, 10713 Berlin, Germany

**Keywords:** Chloroquine induced maculopathy, ABCA4, Age-related macular degeneration, Stargardt’s disease, Genetic association

## Abstract

**Background:**

Chloroquine (CQ) and hydroxychloroquine (HCQ) are used to treat auto-immune related diseases such as rheumatoid arthritis (RA) or systemic lupus erythematosus. Both drugs however can cause retinal toxicity eventually leading to irreversible maculopathy and retinopathy. Established risk factors are duration and dosage of treatment while the involvement of genetic factors contributing to toxic maculopathy is largely unclear. To address the latter issue, this study aimed to expand on earlier efforts by (1) evaluating risk-altering variants known to be associated with age-related macular degeneration (AMD), a frequent maculopathy in individuals over 55 years of age, and (2) determining the contribution of genetic variants in the coding sequence of the *ABCA4* gene.

**Methods:**

The *ABCA4* gene was analyzed by deep sequencing technology using a personal genome machine (Ion Torrent) with 200 bp read length. Assessment of AMD variants was done by restriction enzyme digestion of PCR products and TaqMan SNP genotyping. Effect sizes, p-values and confidence intervals of common variants were evaluated by logistic regression (Firth’s bias corrected). To account for multiple testing, p-values were adjusted according to the false discovery rate.

**Results:**

We found no effects of known AMD-associated variants on the risk of toxic maculopathy. In contrast, we report a statistically significant association of common variants in the *ABCA4* gene with retinal disease, assessed by a score-based variance-component test (P_SKAT_ = 0.0055). This association remained significant after adjustment for environmental factors like age and duration of medication and was driven by three common variants in *ABCA4* (c.5682G > C, c.5814A > G, c.5844A > G), all conferring a reduced risk for toxic maculopathy.

**Conclusions:**

Our findings demonstrate that minor alleles of common genetic variants in *ABCA4* significantly reduce susceptibility to develop toxic maculopathy under CQ treatment. A refined risk profile based on genetic and environmental factors may have implications for revised recommendations in CQ as well as HCQ treatment.

**Electronic supplementary material:**

The online version of this article (doi:10.1186/s12886-015-0008-0) contains supplementary material, which is available to authorized users.

## Background

Chloroquine (CQ) and hydroxychloroquine (HCQ) are chemical compounds frequently used to treat auto-immune related diseases such as rheumatoid arthritis (RA) or systemic lupus erythematosus. Side effects of the treatment include gastrointestinal problems, itching and headaches and, more seriously, depression, cardiomyopathies and CQ-induced retinal toxicity, the latter eventually leading to irreversible maculopathy and retinopathy. The prevalence of CQ-induced maculopathy (herein referred to as toxic maculopathy) has been reported to be between 10% and 25% for patients treated with CQ and between 0.5% and 7.5% after HCQ therapy [1-4]. So far, there is no treatment addressing the drug-related side effects.

The initial symptom of toxic retinal damage is pericentral relative scotoma in the automated threshold perimetry. If the drug is not discontinued at this early phase, the characteristic bulls-eye shaped alterations of the macula develop, leading to irreversibly reduced visual acuity and absolute (peri-)central scotoma.

Recently, potential risk factors for toxic maculopathy due to HCQ treatment have been investigated in a large collection of patients [4]. The authors conclude that duration of use, daily doses of the drug as well systemic kidney disease and concurrent tamoxifen citrate therapy are risk factors for toxic maculopathy. Furthermore, this study proposed to use real body weight instead of ideal body weight to calculate the daily dose of HCQ, since real body weight predicted the risk for toxic maculopathy better than ideal body weight. In an earlier retrospective study including 51 patients treated with CQ or HCQ, age and the duration of intake were suggested as major risk factors for toxic maculopathy [5]. Although a timely diagnosis of toxic retinal damage by adhering to the screening guidelines of the American Academy of Ophthalmology [6] is highly warranted, Nika *et al*. [7] showed that even in a health-insured population many high risk patients are not screened regularly for toxic side effects of the treatment.

So far, there are only few clues as to a contribution of genetic factors to the risk of toxic maculopathy. One study found that two out of eight patients with toxic maculopathy carried monoallelic mutations in *ABCA4* [8], a gene in which mutations have been associated with autosomal recessive Stargardt disease [9] and the genetically complex age-related macular degeneration [10,11]. Interestingly, mutations in *ABCA4* were also implicated to cause hereditary bull’s eye maculopathy (BEM) [12,13], a phenotype reminiscent of toxic maculopathy.

The exact mechanism of retinotoxicity of CQ and HCQ is not clear; however, cell culture studies and animal model experiments suggest a possible adverse effect on lysosomal function and subsequent lipofuscin accumulation [14,15]. Lipofuscin appears to be toxic for photoreceptors as well as RPE cells and may be causal to degenerative processes in retinal diseases such as Stargardt’s disease or age-related macular degeneration (AMD) [16,17].

Here, we expand on previous efforts to evaluate variants in the *ABCA4* gene for their potential contribution to toxic maculopathy [8]. We analyzed the *ABCA* gene in a sample of 37 patients treated with CQ of which 24 had and 13 had not developed toxic maculopathy at the time of the study. Our cohort did not include patients with HCQ-induced maculopathy.

We sequenced the 50 coding exons and the immediate flanking intronic sequences of the *ABCA4* gene and compared the findings to publicly available data obtained in 267 European individuals from the 1000 Genomes Project. Since toxic maculopathy shares phenotypic similarities with AMD such as central macular degeneration and lipofuscin accumulation, we also investigated the impact of genetic risk factors known to be associated with AMD. Therefore, we genotyped toxic maculopathy patients and controls for ten common AMD risk variants and calculated a genetic risk score according to Grassmann *et al*. [18].

Knowledge about genetic factors conferring susceptibility to toxic maculopathy could help to identify patients at greater risk to develop toxic side effects when treated with CQ or HCQ. This could be a first step to an individualized treatment regimen.

## Methods

### Patient recruitment

This study followed the tenets of the Declaration of Helsiniki and was approved by the ethics committee of the Charité (Ethikkommission der Charité – Universitätsmedizin Berlin; date of approval: 12.1.2010; application number: EA2/100/09). All subjects gave informed consent to participate in the study.

Medical records of the patients who presented at the Department of Ophthalmology at the Charite Hospital between 2000 and 2010 were reviewed retrospectively for evaluation of retinal damage due to CQ treatment. To be classified as a *case*, patients had to exhibit unequivocal macular and functional alterations (i.e. bull’s eye maculopathy on funduscopy and/ or optical coherence tomography and/ or fundus autofluorescence imaging, pericentral scotoma in the automated threshold perimetry, pericentrally decreased amplitudes in the multifocal electroretinogram). To be classified as a *control*, CQ-treated patients revealed no signs of macular damage based on any of the aforementioned clinical criteria. Patients which could not be classified unanimously as affected or unaffected by toxic maculopathy or which revealed macular alterations of possibly other aetiology (like age-related macular degeneration) were excluded from the study.

Patients eligible for the study were contacted via phone or mail and asked for participation. Risk factors as postulated by the American Academy of Ophthalmology [6] were inquired. Furthermore, patients on CQ treatment during the period between January 2010 and December 2013 were directly included in the study at the time of presentation at the Ophthalmology Department.

Ophthalmologic findings, data on medication history and risk factors were generally considered at the time of initial presentation or when a definitive ophthalmologic diagnosis was made.

### Genotyping of AMD-associated risk variants

We genotyped ten AMD associated risk variants in six gene loci according to the parsimonic risk model of Grassmann *et al.* [18]. Genotyping of single nucleotide polymorphisms (SNPs) was achieved by restriction enzyme digestion of PCR products (RFLP) and TaqMan SNP Genotyping (Applied Biosystems, Foster City, USA) as described previously [18]. We coded each genotype as number of risk increasing alleles (0, 1 or 2) and computed the risk score with the coefficients obtained from figure one in [18]. The constant “a” was chosen as 8.97, which is the mean genetic risk score calculated from these ten variants in 267 European samples in the 1000 Genomes Project (Release 20110521, http:// www.1000genomes.org, accessed 2 May 2012).

### Sequencing of the ABCA4 gene and data analysis

DNA was extracted as previously described [18]. To amplify the 50 coding *ABCA4* exons and their immediate flanking intronic sequences a custom-made STGD1 MASTR Assay (Multiplicom, Niel, Belgium) was used. The multiplexed PCR fragments of each patient were pooled equimolarly and processed using the Ion Xpress™ Plus Fragment Library kit (Life Technologies) according to the manufacturers recommendations. We used the Ion Xpress™ Barcode Adapters 1–16 to allow for multiplexing of DNA libraries. The resulting DNA libraries were purified with AMPure beads (Beckman Coulter), and their concentrations and sizes were determined on an Agilent BioAnalyzer DNA high-sensitivity Chip (Agilent Technologies). Between 5 and 10 libraries were pooled equimolarly and sequenced in one sequencing run. Emulsion PCR and enrichment of cDNA-conjugated particles were performed with the Ion OneTouch™ 200 Template Kit v2 DL (Life Technologies) according to the manufacturer’s instructions. The final particles were loaded on an Ion 316 chip and sequenced with a Personal Genome Machine (Ion Torrent) with 200 bp read length (Life Technologies).

Reads were aligned to the *ABCA4* reference (NM_000350.2) using the CLC Genomics Workbench (http://www.clcbio.com). Coverage per exon and minimum coverage per coding base were recorded. In order to assure sufficient power to detect variants, the minimum coverage threshold of a coding base was set to 30. Fragments failing this threshold were re-sequenced with the Sanger sequencing chain termination method. Variants were called with the Probablistic Variant Caller in CLC. We excluded called variants in intronic regions beyond 10 bp within the donor and acceptor splice sites, respectively. A genotype was coded as the number of minor alleles at a given variant (0, 1 or 2).

To compare obtained allele frequencies with a larger reference sample, we extracted the genotypes of all variants for 267 EUR samples in the 1000 Genomes Project (Interim Release 20110521) and coded the genotype according to the number of minor alleles.

### Statistical analysis

To evaluate whether variants in *ABCA4* jointly (common and rare; protective and adverse) are associated with toxic maculopathy, the genotype matrix containing the identified *ABCA4* variants was analysed with a SNP-set (Sequencing) Kernel Association Test (SKAT) in R [19] using the functions *SKAT_Null_Model* and *SKAT_CommonRare* with standard settings from the package “SKAT” [20]. We fit two baseline models (null models), one without any covariates (unadjusted model) and one including age, gender, duration of medication (in months) and daily dose per ideal body weight (in mg/kg) as covariates (adjusted model). In order to assess whether rare or common or both variants are responsible for the observed association, we used the options *test.type = “Rare.Only”* and *test.type = “Common.Only”*. The cut-off frequency threshhold for “rare” and “common” was assessed by CommonRare_Cutoff = 1/√(2*sample size).

Logistic regression was used to evaluate the effect size, p-values and confidence intervals of common genetic variants in *ABCA4* as well as AMD associated variants. The association of clinical variables with disease risk was assess by the two-sided student’s t-test. To adjust the association of genetic variants for clinical variables like treatment duration or daily dose per ideal body weight, we used Firth’s bias reduced logistic regression for small sample sizes [21] implemented in the R package logistf [22]. Multiple testing was accounted for by adjusting the observed raw p-values according to the false discovery rate (FDR) [23].

## Results

Twenty-four patients with toxic maculopathy and 13 patients with no signs of maculopathy after CQ treatment were included in the study. A summary characteristics of the patient population is given in Table 1. We first evaluated the differences of clinical risk factors between patients with (cases) and those without (controls) toxic maculopathy after CQ treatment. Cases were significantly older than controls and were exposed to a higher average daily dose per ideal body weight (P_t-test_ < 0.05). Then, we investigated the effect of ten common AMD-associated variants on the risk of toxic maculopathy. The results of the unadjusted logistic regression analysis are summarized in Additional file 1: Table S1 demonstrating that no statistically significant association of known AMD-associated variants with CQ-induced maculopathy was detected. Additionally, a genetic risk score [18] was computed and was −0.43 (SD: 1.32) for cases and 0.20 (SD: 1.06) for controls. This association is statistically not significant (P_t-test_ = 0.148).

**Table 1:** **Summary characteristics of CQ-treated patients**

	Cases*	**Controls** ^**†**^	**p-value (two-sided t-test)**
**Number of Individuals**	24	13	-
**Mean Age (S.D.) [years]**	61.18 (10.45)	49.11 (9.74)	0.0016
**Treatment Duration (S.D.) [months]**	153.1 (97.77)	121.8 (58.74)	0.2327
**Daily dose per body weight (S.D.) [mg/kg]**	4.00 (1.26)	3.90 (0.84)	0.7966
**Daily dose per ideal weight (S.D.) [mg/kg]**	4.90 (1.25)	4.07 (0.31)	0.0078
**Fraction of Individuals > 60 years**	0.71	0.15	-
**Male [%]**	0.04	0.23	0.1622

^*^Patients treated with CQ and affected with toxic maculopathy.

^†^Patients treated with CQ and no signs of toxic maculopathy.

To investigate the contribution of variants in *ABCA4* on toxic maculopathy risk, we sequenced the 50 *ABCA4* exons and their immediate flanking intronic sequences in the 37 patients treated with CQ. In total, we identified 23 *ABCA4* variants in the 37 CQ-treated patients and, after extraction of the genotypes for the 267 EUR samples from the 1000 Genomes project (if present), the frequencies in the three groups were computed (Table 2).

**Table 2:** **Genetic variants identified in** ***ABCA4*** **sequence analysis in CQ-treated patients with (cases) and without (controls) toxic maculopathy**

		**Frequency in**				
**Variant (NM_000350.2)**	**Amino acid exchange (NP_000341.2)**	**Cases**	**Controls**	**EUR** ^**†**^	**Raw p-value**	**FDR** ^**#**^
**c.324G > A**	M114I	0.00	0.04	-	**-**	**-**
**c.635G > A**	R212H	0.06	0.08	0.06	**-**	**-**
c.1268A > G*	H423R	0.29	0.23	0.30	0.58783	0.58783
**c.1269C > T**	H423H	0.13	0.04	0.07	**-**	**-**
**c.1622T > C**	L541P	0.02	0.00	-	**-**	**-**
**c.2588G > C**	G863A	0.00	0.04	0.00	**-**	**-**
**c.2828G > A**	R943Q	0.04	0.12	0.04	**-**	**-**
**c.3113C > T**	A1038V	0.02	0.00	0.00	**-**	**-**
**c.4203C > A**	P1401P	0.00	0.04	-	**-**	**-**
**c.4297G > A**	V1433I	0.00	0.04	0.00	**-**	**-**
**c.5603A > T**	N1868I	0.06	0.08	0.07	**-**	**-**
c.5682G > C*	L1894L	0.13	0.38	0.26	0.02292	0.030
c.5814A > G*	L1938L	0.06	0.31	0.18	0.00722	0.014
**c.5843C > T**	P1948L	0.04	0.08	0.04	**-**	**-**
c.5844A > G*	P1948P	0.06	0.31	0.19	0.00722	0.014
**c.6069T > C**	I2023I	0.04	0.08	0.06	**-**	
**c.6148G > C**	V2050L	0.02	0.00	0.00	**-**	**-**
**c.6249C > T**	I2083I	0.04	0.08	0.05	**-**	**-**
**c.6282 + 7G > A**	-	0.04	0.08	0.05	**-**	**-**
**c.6285T > C**	D2095D	0.08	0.15	0.10	**-**	**-**
**c.6357A > G**	E2119E	0.02	0.00	-	**-**	**-**
**c.6730-3T > C**	-	0.02	0.12	0.02	**-**	**-**
**c.6764G > T**	S2255I	0.02	0.12	0.02	**-**	**-**

*Common variants (combined frequency in cases and controls > 11.6%).

^†^Frequency in 267 European individuals obtained from the 1000 Genomes Project.

^#^False discovery rate.

To assess whether the identified variants in *ABCA4* jointly are associated with toxic maculopathy, we used SKAT on the resulting genotype matrix of variants found in cases and controls. Variants were considered “rare” if the frequency of the variants in cases and controls jointly was lower than 11.6% (i.e. lower than 1/√(2*sample size)). A statistically significant association of this SNP set with CQ-induced maculopathy was found (P_SKAT_ = 0.0055). This association remained statistically significant after adjusting the baseline SKAT model for gender, treatment duration (in months), age (in years) and daily dose per ideal weight (in mg/kg) (P_SKAT_ = 0.025). This effect appears to be driven by common variants (i.e. variants with a frequency above 11.6%). We observed a statistically significant association when restricting the SKAT model to common variants (P_SKAT_ = 0.005 for the unadjusted model, P_SKAT_ = 0.008 for the adjusted model), while we failed to find significance for rare variants (P_SKAT_ = 0.223 for the unadjusted model, P_SKAT_ = 0.755 for the adjusted model). We therefore limited the further statistical evaluation to variants with a combined frequency above 11.6% in cases and controls (indicated by an asterisk in Table 2). This revealed a statistically significant association of three synonymous variants (c.5682G > C, c.5814A > G, c.5844A > G) after adjustment for multiple testing (false discovery rate < 0.05). The frequencies for these variants differed by more than 20% between cases and controls (Table 2) and showed a protective effect, i.e. odds ratios smaller than one (Table 3). We also investigated whether this association was independent of CQ dosage, age and gender and computed logistic regression models for each SNP adjusted jointly for age, gender, duration of treatment and daily dose per ideal body weight. We found a consistent (and significant) protective effect for these variants indicating an independent association with toxic maculopathy (Table 3).

**Table 3:** **Sensitivity analysis of significantly associated variants**

	**Cases vs. Controls**		**Cases vs. EUR** ^**†**^
**Variant**	**OR (95% CI** ^**#**^ **), unadjusted model**	**OR (95% CI** ^**#**^ ), adjusted model*	**OR (95% CI** ^**#**^ **), unadjusted model**
**c.5682G > C**	0.30 (0.09-0.85)	0.05 (0.00 - 1.00)	0.43 (0.16-0.94)
**c.5814A > G**	0.16 (0.03-0.63)	0.01 (0.00 - 0.27)	0.32 (0.08-0.91)
**c.5844A > G**	0.16 (0.03-0.63)	0.01 (0.00 - 0.27)	0.29 (0.07-0.80)

*Adjusted jointly for age, gender, treatment duration and daily dose per ideal bodyweight.

^†^Patients treated with CQ and affected with toxic maculopathy were compared to data from 267 European individuals obtained from the 1000 Genomes Project.

^#^Confidence intervals.

When comparing cases with EUR samples, we again found statistically significant odds ratios smaller than one (Table 3) for all three protective variants. Two variants (c.5814A > G and c.5844A > G) are in high linkage disequilibrium with r^2^ = 0.95 based on the CEU (Northern and Western European ancestry) reference panel in the 1000 Genomes Project (http://1000genomes.org). These variants therefore represent the same risk haplotype and should confer the risk jointly.

The associated variants (c.5682G > C, c.5814A > G, c.5844A > G) represent synonymous alterations with no obvious effect on amino acid composition of the ABCA4 transporter. Two of these (c.5682G > C and c.5844A > G) influence codon usage and each result in about 50% less frequently used codons [24]. Variant c.5682G > C changes the codon from CTG (3.99%) to CTC (1.96%) and c.5844A > G changes the codon from CCA (1.69%) to CCG (0.69%). In contrast, c.5814A > G changes the codon from TTA (0.77%) to TTG (1.29%), a more frequently used codon.

## Discussion

This study aimed to further explore a potential influence of genetic variants on the risk of developing toxic maculopathy after prolonged CQ treatment. First, we evaluated a possible association of known AMD-associated variants on disease risk but found no statistically significant evidence for this hypothesis. However, our analysis can not exclude that other as yet unknown AMD-associated variants may play a role in the etiology of toxic maculopathy or that our patient cohort may lack statistical power to detect significant differences with minor effects.

We then assessed the *ABCA4* gene and identified genetic variants significantly associated with CQ-induced maculopathy. This association is mainly driven by three common variants (c.5682G > C, c.5814A > G and c.5844A > G), whose minor alleles confer increased protection from the toxic maculopathy. While the study provides robust and significant association data, the sample size of 37 patients is relatively small in principle due to the rarity of toxic maculopathy but also to the fact that nowadays CQ is less frequently used for therapeutic purposes than HCQ which possible shows reduced side effects with regard to treatment-induced pathology. Even more challenging is the recruitment of control subjects who received CQ but never developed maculopathy under this treatment. Considering the limited number of available cases and controls in this study, it is obvious that our statistical evaluation had to be limited to common variants while rare variant analysis would have lacked sufficient statistical power. In fact, our study design would theoretically require at least 200 cases and controls to obtain a moderate power of 21% to detect a significant association with a set of rare variants (P_SKAT_ < 0.05 with a minor allele frequency below 0.03).

A comparison to Shroyer *et al.* [8], a study which included a total of only 8 patients suffering from toxic maculopathy upon CQ treatment, showed a deleterious effect of *ABCA4* variants on disease risk and appears to suggest a contradiction to our findings. In this report, however, patient #7 carried three pathologic mutations known to be associated with Stargardt disease (Arg2107His and Leu1201Arg/Arg2107His in a *cis* configuration). As a consequence, this patient may rather be appreciated as manifesting Stargardt disease than toxic CQ-associated maculopathy. Considering the remaining 7 CQ-related maculopathy patients, the frequencies of the minor (in our study protective) *ABCA4* variants are 0.29, 0.14 and 0.14 for c.5682G > C, c.5814A > G and c.5844A > G, respectively. These frequencies are higher than those in our data set in the CQ-associated maculopathy cases but clearly lower than our controls and thus reveal a similar orientation of effect for these three polymorphic alleles.

The odds ratios per *ABCA4* risk allele indicate a strong risk reduction of risk-altering allele carriers towards toxic maculopathy. Two variants in *ABCA4* (c.5814A > G and c.5844A > G) are in close proximity to each other demonstrating strong linkage disequilibrium. It is therefore likely that the two variants represent a common associated (protective) haplotype. A larger patient sample size will be needed to statistically determine the true toxic maculopathy-associated variant, c.5814A > G or c.5844A > G. It should be emphasized that the range of risk reduction of the toxic maculopathy-associated *ABCA4* variants is well comparable to odds ratios of non-risk alleles at the *CFH* or *ARMS2/HTRA1* loci, two genomic regions which are considered to be *strongly* associated with large effect sizes with the development of AMD [25].

Although synonymous variants are commonly regarded as benign in their effects towards disease, they have the potential to affect RNA stability and RNA splicing and can influence the rate of protein translation and thus protein conformation [26-29]. Associations of synonymous variants with complex phenotypes are not unusual and this could call for a paradigm shift when considering the functional impact of these variants on disease and complex traits. Alternatively, other so far unknown variants in linkage disequilibrium with the identified synonymous variants may influence disease risk by altering splicing or by influencing transcription levels due to reduced transcription factor (enhancer) binding. Ultimately, to determine the functional variants at the *ABCA4* locus which could be responsible for a protective effect on toxic maculopathy, more in-depth studies in cellular or animal model systems will be required.

Since our study lacks sample size to reliably evaluate risk scores and project absolute risk estimates, we cannot at present evaluate the combined effect of these genetic variants with or without other risk factors like age or duration of treatment. However, it is expected that these factors together with genetic data will result in an excellent risk prediction accuracy and therefore be deemed important to be evaluated in a larger study despite the given constraints on patient recruitment as discussed above. For instance, the National Eye Institute (NEI) is currently recruiting patients for a clinical trial (NCT01145196) to assess the impact of *ABCA4* mutations on toxic maculopathy risk.

Should this knowledge of protective genetic variants have an influence on clinical practice? The *ABCA4*-analysis in any patient who is about to receive CQ would generally be desirable. As this analysis is not covered by the health insurance the patient himself has to bear the financial burden. Therefore, if recommended, it should most likely be done primarily in CQ patients with a non-genetic high-risk profile, i.e. in older patients with a high dose per body weight, high body mass index, restriction of excretory liver and renal function or in patients whose eyesight is reduced due to other reasons. If the combined genetic and non-genetic profile indicates a high risk, another disease-modifying drug might be preferred. In principle, phenotypes and non-genetic risk factors of CQ and HCQ maculopathy are similar. Therefore, the results of the present study may be projected onto HCQ maculopathy. A recent study found an overall prevalence of 7.5% for HCQ-induced maculopathy [4], which further emphasizes the medical need to treat patients based on an individual risk profil for possible side effects. Further and larger sized studies will be needed to replicate the current findings, specifically in patients treated with HCQ, a drug nowadays replacing CQ.

## Conclusions

Taken together, our findings demonstrate that minor alleles of common genetic variants in *ABCA4* significantly reduce susceptibility to develop toxic maculopathy under CQ treatment. A refined risk profile based on genetic and environmental factors may have implications for revised recommendations in CQ as well as HCQ treatment. As our approach was targeted to include candidate genes upon a sophisticated guess as to their possible relation with the disease, it would be desirable to extend the genetic analyses to a genome-wide unbiased screening approach. This would allow to decipher the full genetic spectrum of CQ-and hopefully HCQ-induced maculopathy.

## Additional file

Additional file 1: Table S1. Association results for 10 known AMD associated variants using single logistic regression in patients treated with chloroquine.
