# Supplementary material for: Common synonymous variants in ABCA4 are protective for chloroquine induced maculopathy (toxic maculopathy)
Source: BMC Ophthalmol. 2015 Mar 6;15:18. doi: 10.1186/s12886-015-0008-0 (PMC4352241; doi:10.1186/s12886-015-0008-0)
Supplement: Additional file 1: Table S1. — Association results for 10 known AMD associated variants using single logistic regression in patients treated with chloroquine. [file 12886_2015_8_MOESM1_ESM.docx]

|  |  |  |  |  |  | Frequency of risk allele in | |
| --- | --- | --- | --- | --- | --- | --- | --- |
| Variant | **Impact/effect of variant** | **Odds ratio**  **(95% CI*)** | **P-value**^†^ | **Non risk allele** | **Risk allele**^‡^ | **cases^§^** | **controls^&^** |
| C3_rs2230199 | p.R102G | 0.424  (0.09-1.938) | 0.264 | G | C | 0.130 | 0.227 |
| ARMS2_rs10490924 | p.A69S | 1.082  (0.329-4.175) | 0.899 | G | T | 0.196 | 0.182 |
| CFB_rs4151669 | proxy for rs4151668 (p.L9H) | 1.029  (0.068-7.283) | 0.977 | A | T | 0.957 | 0.955 |
| CFH_rs1061170 | p.Y402H | 1.067  (0.287-4.109) | 0.922 | T | C | 0.283 | 0.273 |
| CFH_rs800292 | p.I62V | 0.852  (0.251-2.541) | 0.779 | A | G | 0.739 | 0.773 |
| CFH_rs6677604 | proxy for ∆CFHR3/CFHR1 | 0.294  (0.057-1.111) | 0.100 | A | G | 0.674 | 0.864 |
| CFB_rs438999 | proxy for rs641153 (p.R32Q) | NA | 0.995 | C | T | 0.891 | 1.000 |
| APOE_rs7412 | p.R158C | 1.250  (0.219-9.934) | 0.811 | C | T | 0.109 | 0.091 |
| APOE_rs429358 | p.C112R | 0.349  (0.048-1.531) | 0.213 | C | T | 0.783 | 0.909 |
| PLA2G12A_rs2285714 | synonymous, unknown | 1.090  (0.428-2.878) | 0.856 | C | T | 0.435 | 0.409 |

**Additional file 1: Table S1** Association results for 10 known AMD associated variants using single logistic regression in patients treated with chloroquine

^*^CI = confidence interval; ^†^P-values were derived from a logistic regression model with one SNP as covariate; ^‡^Risk allele is the allele that is associated with increased risk of AMD; ^§^cases denotes patients with toxic maculopathy upon treatment with chloroquine; ^&^controls denotes patients treated with chloroquine but no signs of toxic maculopathy
